# Deep Brain Stimulation and L-DOPA Therapy: Concepts of Action and Clinical Applications in Parkinson's Disease

**DOI:** 10.3389/fneur.2018.00711

**Published:** 2018-08-27

**Authors:** Muthuraman Muthuraman, Nabin Koirala, Dumitru Ciolac, Bogdan Pintea, Martin Glaser, Stanislav Groppa, Gertrúd Tamás, Sergiu Groppa

**Affiliations:** ^1^Movement Disorders and Neurostimulation, Biomedical Statistics and Multimodal Signal Processing Unit, Department of Neurology, University Medical Center of the Johannes Gutenberg University, Mainz, Germany; ^2^Department of Neurology, Institute of Emergency Medicine, Chisinau, Moldova; ^3^Laboratory of Neurobiology and Medical Genetics, Nicolae Testemiţanu State University of Medicine and Pharmacy, Chisinau, Moldova; ^4^Department of Neurosurgery, University Hospital of Bonn, Bonn, Germany; ^5^Department of Neurosurgery, University Medical Center of the Johannes Gutenberg University, Mainz, Germany; ^6^Department of Neurology, Semmelweis University, Budapest, Hungary

**Keywords:** Parkinson's disease, levodopa, deep brain stimulation (DBS), subthalamic nucleus (STN), globus pallidus internus (GPi)

## Abstract

L-DOPA is still the most effective pharmacological therapy for the treatment of motor symptoms in Parkinson's disease (PD) almost four decades after it was first used. Deep brain stimulation (DBS) is a safe and highly effective treatment option in patients with PD. Even though a clear understanding of the mechanisms of both treatment methods is yet to be obtained, the combination of both treatments is the most effective standard evidenced-based therapy to date. Recent studies have demonstrated that DBS is a therapy option even in the early course of the disease, when first complications arise despite a rigorous adjustment of the pharmacological treatment. The unique feature of this therapeutic approach is the ability to preferentially modulate specific brain networks through the choice of stimulation site. The clinical effects have been unequivocally confirmed in recent studies; however, the impact of DBS and the supplementary effect of L-DOPA on the neuronal network are not yet fully understood. In this review, we present emerging data on the presumable mechanisms of DBS in patients with PD and discuss the pathophysiological similarities and differences in the effects of DBS in comparison to dopaminergic medication. Targeted, selective modulation of brain networks by DBS and pharmacodynamic effects of L-DOPA therapy on the central nervous system are presented. Moreover, we outline the perioperative algorithms for PD patients before and directly after the implantation of DBS electrodes and strategies for the reduction of side effects and optimization of motor and non-motor symptoms.

## Introduction

The principal pathological characteristic of Parkinson's disease (PD) is the progressive death of the pigmented neurons of the substantia nigra pars compacta (SNc) diagnosed by symptoms including bradykinesia/akinesia, rigidity, postural abnormalities and tremor (1). The discovery in the 1960s that the degeneration of the dopamine (DA) neurons of the SNc cause parkinsonism (2) prompted the development of pharmacological therapies for PD using the DA precursor L-3,4-dihydroxypheylalanine (L-DOPA or levodopa) to enhance synaptic DA transmission (3). Five decades after its introduction, L-DOPA is still the most effective and widely used drug to alleviate the symptoms of PD (4). In recent years, deep brain stimulation (DBS) has become a standard evidence-based therapy for severe movement disorders such as PD (5), tremor (6) and dystonia (7). Since the first DBS surgery in Grenoble nearly 30 years ago (8), over 100,000 patients have undergone DBS implantations for neurologic and neuropsychiatric conditions (9). Even though DBS has been investigated for more than 20 different clinical indications and 40 distinct targeted areas (10), the mechanisms through which DBS modulates the underlying brain networks and the effects of local stimulation on brain functioning are still poorly understood (11–13). Whether DBS suppresses or activates local neuronal elements, interrupts or modulates the information flow within the cerebral networks (14–16) or improves the signal-to-noise ratio in a stochastic system (17) is still a matter of debate.

Medically intractable motor fluctuations and tremor are independent indications for DBS in PD, in which the electrodes are most commonly implanted in the subthalamic nucleus (STN) or globus pallidus internus (GPi) (5). Bilateral STN-DBS effectively improves the motor fluctuations, bradykinesia and tremor (18). Bilateral GPi stimulation has analogous effects on these symptoms (19), except that comparable tremor relief is less likely to be achieved with this implantation site (20). DBS of the thalamic ventral intermediate nucleus (VIM) is a less common alternative target in patients with tremor-dominant PD, refractory to medication (21). In this review, we outline the similarities and differences in dopaminergic treatment and DBS on neurophysiological, anatomical and clinical levels. Based on this, we discuss how these therapies should be efficiently superimposed in the long-term to achieve an optimal clinical outcome.

## Molecular mechanisms of L-DOPA

**Figure 1:**
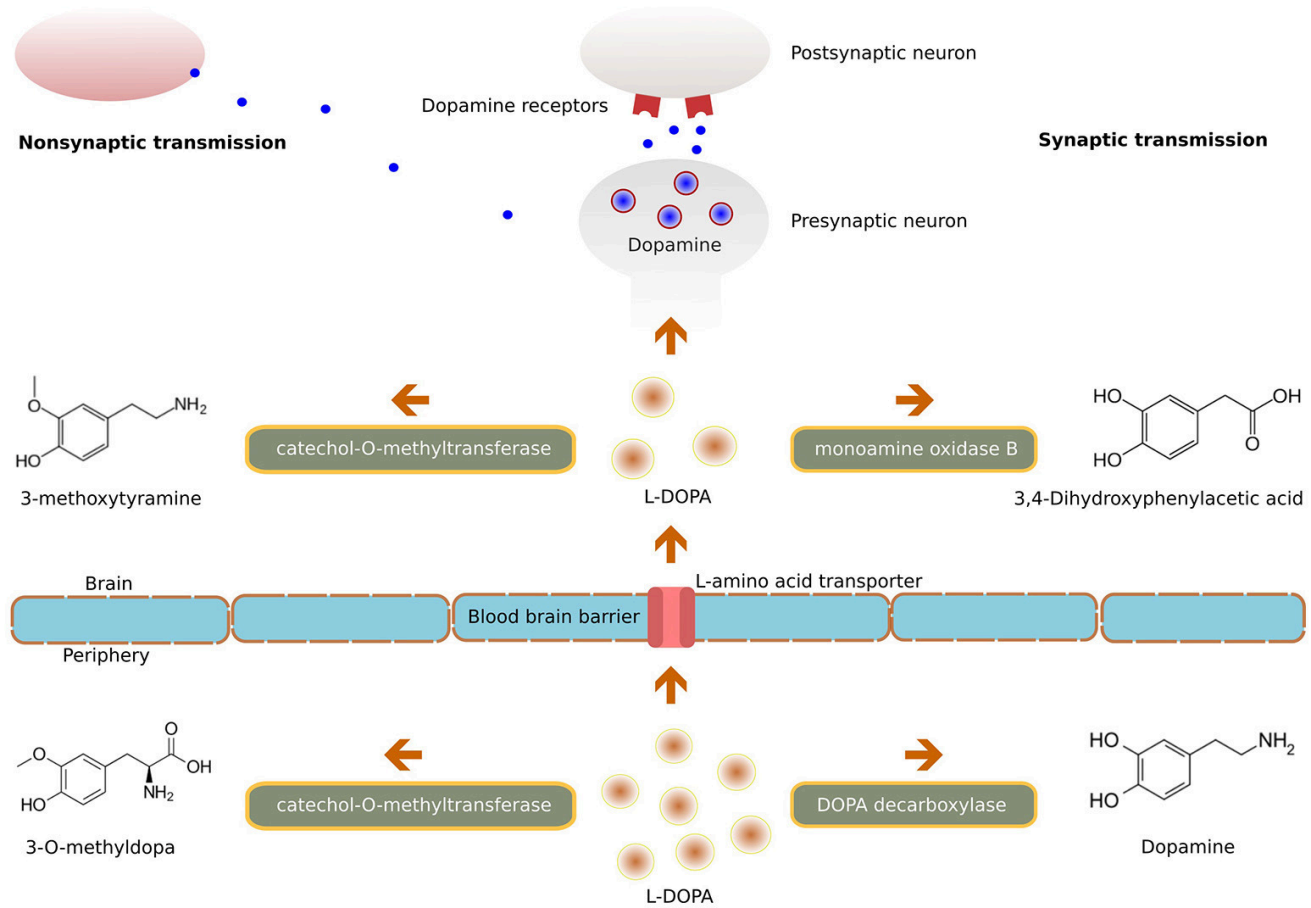
Illustration of the molecular mechanism of L-DOPA.

Parkinsonian symptoms appear when brain levels of dopamine are reduced by 70–80% (22). Dopamine itself has low bioavailability and does not cross the blood-brain barrier (BBB), hence its precursor L-DOPA is used clinically; it is readily transported into the central nervous system (CNS) and is converted into dopamine in the brain by the enzyme DOPA decarboxylase (Figure 1). Only a small quantity of systemically administered L-DOPA enters into the brain; however, this quantity is enough to restore the nigrostriatal dopaminergic neurotransmission. Although conversion into dopamine is the basic mechanism of levodopa's pharmacological effect, it also possesses a direct neuromodulatory action and contributes to the therapeutic efficiency.

L-DOPA is given with carbidopa or benserazide, drugs which do not cross the BBB but inhibit DOPA decarboxylase in peripheral tissues (23). This association of L-DOPA with DOPA decarboxylase inhibitors minimizes it's peripheral degradation, thus extending its half-life (and increasing the availability to the brain) and thereby prolonging the duration of its symptomatic effect (24). Additionally, this reduction in peripheral conversion to dopamine also minimizes the predominant side effects of circulating dopamine like nausea, vomiting and hypotension (25). Another method for improving the bioavailability of L-DOPA is to inhibit its peripheral metabolism via the catechol-O-methyltransferase (COMT) pathway. Inhibition of COMT by tolcapone, entacapone (26) or by a newly available opicapone (27) leads to decreased plasma concentration of 3-O-methyldopa, increased uptake of levodopa, and increased concentrations of dopamine in the brain.

## Effects of dopamine on brain networks

Dopamine is produced in the ventral tegmental area and pars compacta of SN and acts through anatomically segregated but functionally connected pathways (28). The mesocortical, mesolimbic and nigrostriatal dopaminergic systems play a key role in cognition, reward and motor control functions, respectively; their close interaction results in goal-directed behaviors (29).

The midbrain dopaminergic systems project mainly to the striatum and the cortex but also to the thalamus, amygdala, hippocampus and globus pallidus (GP). Its afferent innervation arrives from the striatum and the pedunculopontine nucleus (28). Two subclasses of dopaminergic receptors have been identified in the brain: the D1-like receptor (D1R and D5R) and the D2-like receptor (D2R, D3R and D4R). D1R and D2R are expressed on the GABAergic medium spiny neurons in the dorsal striatum. The D1R contributes to the information flow of the direct pathway, while the D2R contributes to the indirect pathways (30). Their reorganization in the basal ganglia such as increased D2R and decreased D1R expression (31) together with the loss of presynaptic D2R leads to the primary symptoms of PD (30). The highest densities of both D1R and D2R were found in areas that receive a dense dopaminergic innervation such as in nucleus caudatus, GP and putamen in the human brain (32). The SN contains a higher concentration of D1R than D2R. Both receptor types are also expressed in the hippocampus, but only D1R is expressed in amygdala and the neocortex (33). Furthermore, D3R was found in the hypothalamus, in addition to the SN, ventral pallidum/substantia innominata, ventral striatum, GP and thalamus, showing the clear involvement of basal ganglia dopamine circuit (34). In addition, D1, D2 and D3 receptors were identified in the STN and were shown to mediate the effect of dopamine on STN neuronal activities (35).

The combination of different dopaminergic drugs is beneficial, considering their diverse receptor affinity profiles. While dopamine has the highest affinity to D1R, dopamine agonists target mainly D2R. Dopamine agonists can be subdivided into ergoline and non-ergoline derivatives (36). Among the ergoline dopamine agonists, apomorphine is a combined D1R and D2R agonist with more affinity to D2R/D3R than to D1R. In the non-ergoline group, pramipexole, ropinirole and rontigotine are the most widely used dopamine agonists. Pramipexole and ropinirole are known to have a higher affinity to D3R than to D2R, whereas rotigotine is considered to have affinity at several dopamine receptors with a predilection to D1R, D2R and D3R in comparison to D4R and D5R (37). The rare but severe side effects of the ergot-derived dopamine agonists such as fibrosis of the cardiac valves, pleuropulmonary and retroperitoneal fibrosis have limited their use in the clinical practice (38). It has been shown that dopamine is a neurotransmitter that acts not only through synaptic transmission but also by non-synaptic communication (39). In the latter case, dopamine diffuses into the extracellular space and exerts its effect on high-affinity receptors, which are as well-targeted by various drugs (e.g., imipramine).

Administration of L-DOPA increases the functional connectivity within the motor network comprising the putamen, anterior cerebellum and ventral brainstem and ameliorates the motor performance in both healthy (40) and PD populations (41). At the same time, it also decreases the connectivity of the STN-thalamo-cortical motor network (42). The modulatory effects of L-DOPA on motor networks differ among PD patients with different motor subtypes: L-DOPA increases the effective connectivity between posterior putamen and distributed motor network during a tapping task in tremor-dominant PD but not in the postural instability/gait difficulty subtype (43). L-DOPA also increases the coupling between the prefrontal cortex and supplementary motor area (SMA) during a simple motor task (such as finger tapping) but not during tasks requiring higher motor control, hinting at the effect of dopaminergic medication on selective motor control and partial effects on bradykinesia (44, 45). Another study showed that acute levodopa administration significantly enhances the spontaneous functional connectivity in the sensorimotor network in drug-naive patients with PD (46). Taken together, these studies indicate the selective improvement of hypokinetic and bradykinetic movement abnormalities in PD with L-DOPA administration. L-DOPA increases the functional connectivity between the regions related to the cognitive network–the inferior ventral striatum and ventrolateral prefrontal cortex in healthy subjects (40) and in parkinsonian patients (47), in whom maintenance of the working memory performance requires recruitment of the right fronto-parietal network which is as well-boosted by L-DOPA intake (48).

The effect of L-DOPA on cortical networks has been studied considering the modulatory effects of dopamine in basal ganglia. The abnormalities in M1 excitability and plasticity have been demonstrated by several transcranial magnetic stimulation (TMS) based neurophysiological studies (49, 50). Even though there are inconsistencies among studies regarding the findings in altered motor cortical plasticity, almost all of them agree that those abnormalities are improved following L-DOPA administration (51–53). There are also conflicting findings in the PD studies using the paired associative stimulation (PAS) technique revealing abnormalities in M1 long-term plasticity. PAS involves pairing a stimulus to the median nerve (at the wrist) with a TMS pulse given some milliseconds later over M1 (54). Some of these studies demonstrated decreased responses to PAS in patients off treatment, with a partially restored response when on treatment (55). Others showed either no effect of L-DOPA on PAS (56) or an increased response to PAS in PD patients off therapy and restored responses when they were on therapy (57). These differences might be explained by the asymmetric motor symptoms and hemispheric difference in the PAS-induced plasticity (58). Investigation of the mechanism of action of L-DOPA by means of various neurophysiological approaches has revealed that L-DOPA does not restore movement abnormalities, such as the sequence effect or facial bradykinesia but inhibits abnormal neuronal oscillations in basal ganglia, improves reduced discriminative capacities of the sensory system and partially normalizes motor cortex excitability (59, 60). Studies that integrate sensory temporal processing and movement execution for observing the effect of L-DOPA on plasticity and connectivity between the primary motor area and other non-motor cortical areas are still needed.

## Mechanisms of DBS action

Even though the exact underlying physiological mechanism of DBS remains unclear, the therapeutic benefits of DBS seem to be frequency-dependent and can modulate cortical activities. Several animal studies have shown support for the hypothesis of direct cortical activation during STN-DBS (61, 62). These studies have provided evidence of the occurrence of antidromic spikes in M1 during the DBS paradigm which coincides with the optimal effect of STN-DBS. Whether a similar antidromic activation of the known cortex-GPi projection (63) contributes to the therapeutic effect of GPi-DBS remains to be studied. Non-invasive brain stimulation studies using TMS have shown abnormal motor cortical plasticity in PD which has been investigated further for understanding the mechanism of DBS. It has been shown that paired associative cortical plasticity could be induced by repeated STN and M1 stimulations at specific intervals, signifying that STN-DBS can modulate cortical plasticity (64). Moreover, STN stimulation with clinical efficacy increased the excitability of the motor cortex at specific short and medium latencies, suggesting that cortical activation could be one of the mechanisms mediating the clinical effects of STN-DBS in PD (65). It has been further suggested that enhancement of inhibitory synaptic plasticity, non-specific synaptic depletion and frequency-dependent potentiation might be complementary mechanisms of DBS action (66, 67).

**Figure 2:**
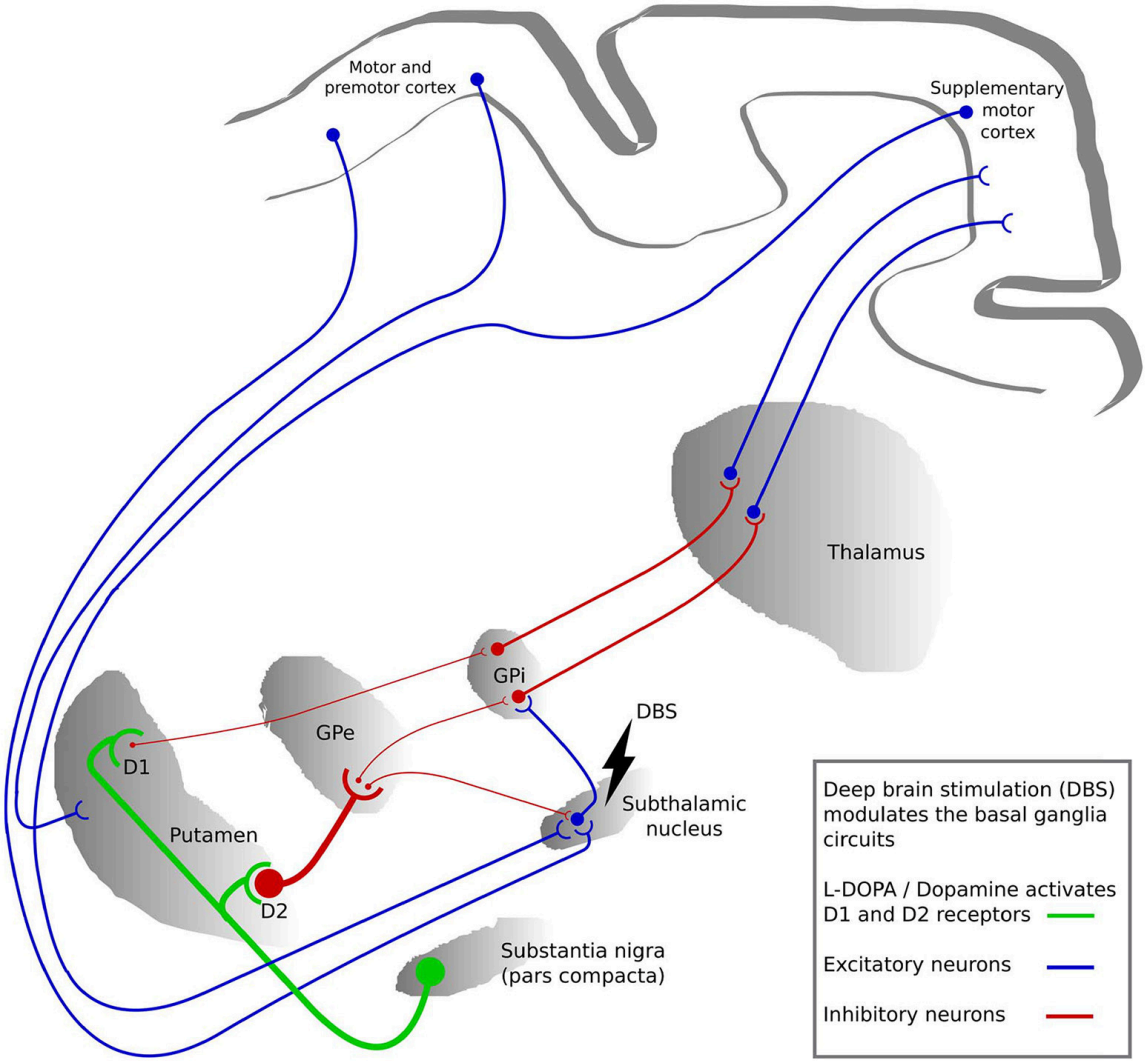
The basal ganglia circuits modulated by deep brain stimulation (DBS) and L-DOPA. The colors represent the activation by L-DOPA (green), excitatory neuronal pathways (blue) and inhibitory neuronal pathways (red). The thickness of the lines between the regions represents the strength of signaling. GPi, globus pallidus internus; GPe, globus pallidus externus.

Several hypotheses exist regarding how DBS acts on neural elements (Figure 2). These hypotheses can broadly be divided into three main categories. The most prevalent is the *suppression hypothesis* in which DBS is supposed to suppress the activity in local neuronal cells and modulate the pathways connecting subcortical and cortical structures, thus having a similar effect as lesioning (68, 69). Inhibition of the neural action potential during stimulation was recorded around the stimulation sites of STN-DBS in patients (70, 71), as well as in monkeys with induced parkinsonism (72, 73). In addition, the complete blockage of neuro-axonal transmission of some STN neurons and the residual neuronal activity of the remaining STN neurons was shown to support the hypothesis of an inhibitory influence of STN-DBS on neuronal activity in STN (74).

The second hypothesis is the *activation* of local neuronal elements. STN-DBS increased neural activity in the interconnected structures such as GPi and substantia nigra (SN) neurons in parkinsonian monkeys (75). In rats, low intensity STN-DBS induced GABAergic suppression in SN through activation of the globus pallidus externus (GPe) neurons, while high intensity STN-DBS induced glutamatergic excitation in the SN (76, 77). Furthermore, it has been shown in rats that STN-DBS induces an increase in activity in motor cortex neurons (62, 78, 79). Another study using optogenetics additionally showed that selective stimulation of cortico-STN afferent axons without activation of STN efferent axons ameliorated the symptoms of parkinsonian mice (61).

The third hypothesis is the *interruption hypothesis* in which the abnormal information flow through the STN is disturbed by STN-DBS. The reciprocal GPi-STN connection produces abnormal synchronized neuronal activity patterns in PD, and this interruption of information flow through the STN reduces these patterns (14, 76). In addition, due to loss of dopaminergic modulation, reduced activity in the striato-GPi direct pathway and hyperactivity along the hyperdirect and striato-GPe indirect pathways is observed in PD. The STN-DBS may disrupt this neuonal activity in STN on the direct and indirect pathways and subsequently reduces such pathological activity (80). Hence, overall STN-DBS might effectively alter the rhythmic interaction by modulating or suppressing the pathological excitability without clearly inhibiting or activating the neural elements (81).

## Network effects of DBS

**Figure 3:**
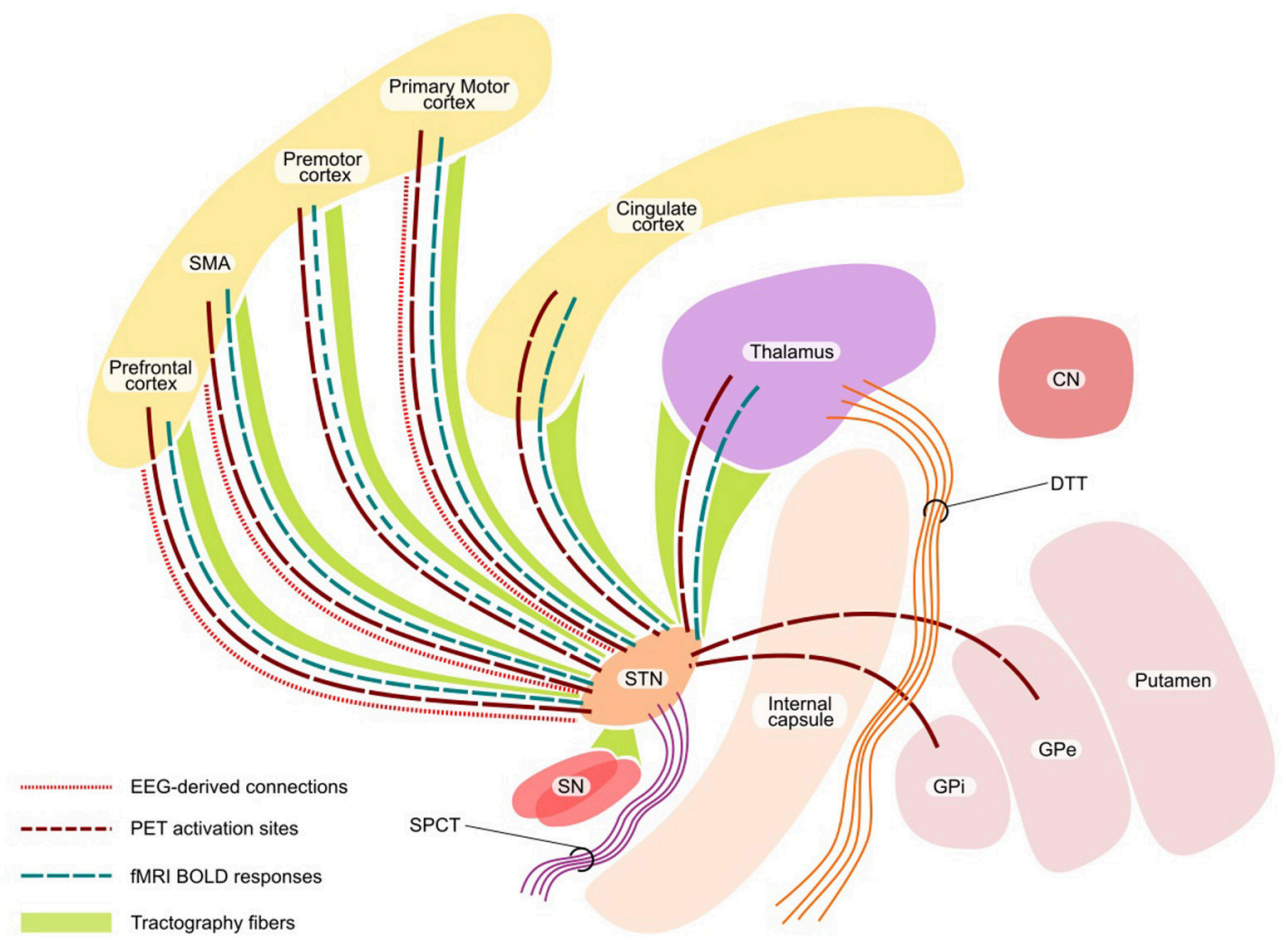
Network effect of DBS. The figure shows the functional network (with PET, fMRI, and EEG) and structural network with tractography. STN, subthalamic nucleus; SN, substantia nigra; CN, caudate nucleus, SMA, supplementary motor area; SPCT, Subthalamo-ponto-cerebellar tract; DTT, Dentate-thalamic tract.

A network perspective on brain structure and function, accounting for the interaction and anatomical connections between regions, offers a potentially valuable framework for the study of physiological brain functioning and for identification of relevant pathological abnormalities at the systemic level. The involvement of STN and GPi in multiple circuits connecting the basal ganglia and cortical regions that mainly regulate the motor, limbic and associative functions is well-established (82–86). This involvement of STN and GPi in various circuits has motivated network-based exploration using various methods for understanding the DBS modulation mechanisms (Figure 3).

### Functional network effects

A considerable number of positron emission tomography (PET) studies have analyzed the functions of the motor system in PD and its relation to DBS treatment. Both resting state and motor task paradigms were investigated using regional cerebral blood flow (rCBF) measurements (87, 88) or by quantifying glucose metabolism (89, 90). These studies showed a decreased activation during STN stimulation at resting state in comparison to no stimulation state in premotor cortex (PMC), dorsolateral prefrontal cortex (DLPFC), SMA and anterior cingulate cortex (ACC) and increased metabolism during task in DLPFC, rostral SMA and ACC (88). Additionally, during the comparison of STN-DBS ON vs. OFF state, increased rCBF was found in midbrain regions (including STN), GP and thalamus (91). Furthermore, several studies addressed cerebral metabolic dynamics during DBS while performing various cognitive tasks. Irrespective of task (motor imagery of gait or stance), during the STN-DBS stimulation OFF condition the activity in sensorimotor cortex, SMA and cerebellum is increased, while during the stimulation ON condition, bilateral STN, precuneus, inferior parietal cortex and cerebellum are activated (92). During both STN-DBS conditions, imagery of gait increased the neural activity in SMA and superior parietal lobule (93). The improvement of imagined gait during the STN-DBS ON condition was linked to the increase in rCBF in the pedunculopontine nucleus/mesencephalic locomotor region without significant modulation of cortical and cerebellar locomotor areas (92). Although the neural correlates of GPi stimulation in PD have been less investigated, some studies have shown an increase in ACC and SMA activation during a motor task (94, 95). These findings were similar to SMA activity modulation during STN-DBS, however in contrast to GPi-DBS there was no increase in DLPFC activation which could be explained by different pathways affected by stimulating each target (96). Furthermore, Fluorodeoxyglucose-positron emission tomography (FDG-PET) measuring resting regional cerebral glucose utilization during ON and OFF phases of stimulation in GPi has shown a reduction in regional metabolic patterns (largely in primary motor cortex) during ON phase which was significantly correlated with the clinical improvement (97, 98).

Studies in STN-DBS patients using functional MRI (fMRI) are limited because of safety concerns and imaging artifacts (99, 100). Nonetheless, recent MR studies performed under specific experimental conditions with resting state and task-based fMRI have shown interesting results. In primates, a recent study provided evidence that STN-DBS significantly increased blood oxygenation level-dependent (BOLD) activation in sensorimotor cortex, SMA, caudate nucleus, pedunculopontine nucleus, cingulate, insular cortex and cerebellum (101). Similarly, a study in rats also found increased BOLD responses in ipsilateral cortical regions, including motor cortex, somatosensory cortex and cingulate cortex during STN- and GPi-DBS (102). No clear patterns of BOLD signal modulation have been tracked during STN-DBS in humans (103), while several common regions have been identified with distinct activation patterns in most of these studies—encompassing ipsilateral thalamic nuclei, SMA, DLPFC, lateral premotor cortex and ACC–corresponding to the networks obtained in animal models and PET studies (104–106). In a study to trace DBS-induced global neuronal network activation in an animal model using fMRI, GPi-DBS activated a larger area of the motor network in comparison to STN-DBS (107). Although STN and GPi stimulation showed common sensorimotor network activation, each was found to activate a distinctive neural network.

### Structural network effects

Even though the target for DBS consists of gray matter structures, DBS predominantly activates the axons rather than cell bodies (108, 109). Hence, study of white matter tracts near the deep nuclei is of great relevance. The measures of white matter microstructural properties and their alterations in various regions of the brain have shed light on important aspects of PD-related pathological process using diffusion imaging (110–112). In the case of PD patients, two tracts, namely the subthalamo-ponto-cerebellar tract (SPCT) and the dentate-thalamic tract (DTT) were identified using probabilistic tractography and showed that active contact positions in proximity to DTT are associated with tremor improvement during the stimulation (113). It has been recently shown using probabilistic tractography that from STN the areas which are frequently connected with the clinically effective contacts included thalamus, SN, brainstem and superior frontal gyrus; the strength of connectivity to the superior frontal gyrus and thalamus correlated with the clinical effectiveness (114). In addition, the modulation of the hyperdirect pathway between the STN and cortical regions was postulated (115). A recent study using diffusion MRI-based tractography (both deterministic and probabilistic) showed that the connections from ipsilateral motor cortex primarily terminated in the dorsolateral STN, further highlighting a key role of hyperdirect pathways in mediating the effects of DBS (116). A diffusion tensor imaging (DTI) analysis investigating the connectivity map of GPi revealed the anterior part to be mainly connected to the prefrontal cortex, the middle section to the brainstem and GPe and the dorsal GPi mainly to the thalamus and GPe (117). In addition, the postero-ventro-lateral part of the GPi, shown to have the most effective clinical outcomes for PD (118), was found to be connected to the thalamus via the pallidothalamic tract. This is further supported by previous findings of the functional perception of the pallidothalamic tracts as the main sensorimotor GPi efference tract (119). One of our recent works pointed out the role of the properties of the targeted network, its connectivity profile and relation to clinical response (86). The topological properties (derived from probabilistic tractography of preoperative MRIs) of the network, involving frontal, prefrontal cortex and cingulate gyrus were directly associated with the post-operative clinical outcome. Particularly, eccentricity (a network measure of the extent of cerebral regions' embeddedness in relation to distant areas) inversely correlated with the DBS stimulation voltage at the active electrode for an optimal clinical response (86). Thus, we could show that the connectivity pattern and topological organization of the DBS-targeted network are important and independent predictors of the post-DBS therapeutic efficiency.

## Effects of dopamine and DBS on brain oscillations in PD

Oscillations at multiple frequencies may reflect information processing in the neural networks (120). It has already been demonstrated that movement-related spectral changes in the activity of the SMA, primary sensorimotor cortex and the basal ganglia indicate a different kinetic state in the patient. In the sensorimotor cortex, elevated synchronization in the beta frequency (15–30 Hz) band was associated with the slowing of voluntary finger movement in healthy subjects (121). Local field potentials (LFPs) of STN in parkinsonian patients undergoing DBS surgery exhibited increased beta activity in the basal ganglia and cortical regions, and correlated with the OFF phase symptoms (122), except tremor (123). This beta activity was found to be decreased after levodopa intake (124). Beta activity in the STN of PD patients is not permanently increased but occurs rather in so-called beta bursts; the amplitude of beta bursts (as the indicator of degree of local neural synchronization) is progressively enhanced with the duration of bursts (125). During the levodopa ON condition, beta bursts become shorter with lower amplitude that correlates with the motor improvement. However, long duration beta bursts are associated with an increase not only in local synchronization but also in interhemispheric synchronization that compromises the patterns of motor processing (125) and is reversed by levodopa treatment. Beta activity is differentially modulated by adaptive and conventional DBS techniques (126). Adaptive DBS elicits a shift from long beta bursts with high amplitude toward shorter bursts with lower amplitude (due to precocious cancelation of long beta bursts by triggered DBS stimulation), while conventional DBS only suppresses beta activity on a global level, without altering the frequency and duration of beta bursts. The increased frequency of short duration beta bursts by adaptive DBS partly explains the mechanisms by which adaptive DBS induces the improvement of motor performance (126).

An excessive synchronization in beta band was identified between the basal ganglia and the motor cortex in PD patients (127, 128). In addition, STN spiking has also been shown to be synchronized with cortical broadband gamma, which occurs in a phase-modulated pattern and begins prior to the occurrence of STN spikes (129). The gamma frequency band (>30 Hz) synchronization in the motor cortex areas and STN of PD patients is thought to represent a prokinetic state which promotes movement-related processing (130, 131). Another physiological phenomenon on the scalp level, the beta band cortico-muscular coherence, seems to be also disturbed in PD with its frequency shifted to the lower ranges in the OFF state and is reversed after levodopa intake (132). It has become evident that oscillatory activity across multiple circuits and frequency bands may be important in the pathophysiology of PD (133, 134). This is perhaps best exemplified by recent simultaneous magnetoencephalography (MEG) and STN-LFP measurements in patients undergoing DBS surgery that demonstrated the existence of multiple regions, which are spatially and spectrally segregated in the STN-cortical oscillatory networks. At rest, a beta band network exists between the STN and motor/premotor areas in addition to a diffuse alpha band network between the STN and temporoparietal regions as well as the brainstem (135). A gamma band network between the STN and motor/premotor cortical areas also intensifies around the time of movement, particularly with dopaminergic therapy (136, 137). With regard to the identified network, it is interesting to note that activities in this frequency band have been linked to orienting attention at a cortical level (138) and also the directionality of this network is predominantly from the cortex toward the STN (136). Although formal confirmation of a putative role for the STN-cortico-brainstem network in orienting attention is still needed, it is worth mentioning that movement-related reductions in coherence in this network (on and off levodopa) correlate with clinical motor improvement (131). Given the above findings, we might speculate that the coupling changes within the STN-cortico-brainstem network in PD may be related to attentional deficits to motor impairment. This hypothesis is supported by previous correlations between attentional deficits in PD and signs of motor impairment such as gait freezing and falls (139, 140). It is intriguing that the levodopa dependence on frequency changes with the reactivity of STN-cortical coupling during voluntary movement, which triggers the bursts of cortical multiunit activity at beta rhythms driving the STN-LFP oscillations (131, 141). The modulation of the amplitude of the cortical broad gamma oscillations is involved in phase amplitude coupling (PAC), which is decreased by DBS, correlating with the motor improvements after DBS (142). In addition, new studies have shown that DBS not only suppresses the elevated resting broadband gamma activity present in PD (143) but that an adaptive DBS could also focus on narrowband gamma oscillations for the reduction of dyskinesia. It has been further proposed that the striatal mechanisms of levodopa-induced dyskinesia (144) might have some links with the gamma oscillations (145).

The resting tremor in PD is thought to be driven by certain oscillators in the brain (146–148). But the neural basis of these tremor oscillations is not very clear as the hand tremor displays a marked spatiotemporal pattern which makes the tremor activities of different limbs almost never coherent (149–151). MEG and electroencephalography (EEG) studies have allowed the characterization of brain regions coherent with parkinsonian resting and postural tremor, hence, revealing the functional tremor networks. These studies have demonstrated the presence of strong electromyogram (EMG) coupling with the signal of the contralateral primary motor cortex (M1) and also cortico-cortical coupling between M1 and other premotor, SMA, somatosensory areas, diencephalic and cerebellar sites (151–153). On the subcortical level, oscillatory peaks at the tremor frequency and its harmonics were revealed within the STN, GPi and thalamus (154–156) in addition to coherence between these sites and the EMG activity (157, 158). Recent work has shown that in the distinct group of brain regions acting synchronously, segregated tremor clusters may relate to tremor activity in specific muscle groups, pointing to multiple tremor-related subloops within subcortical structures (158), which suggests the possible existence of multiple tremor oscillators within the basal ganglia-thalamo-cortical circuits.

## Clinical aspects of dopaminergic therapy

The clinical benefit of levodopa has not been doubted since its description (159) and is still the most effective symptomatic drug for PD (160). Current therapeutic approaches rely upon levodopa and dopamine agonists. Levodopa, the precursor of dopamine moves across the BBB and combines with the peripherally-acting decarboxylase inhibitors such as benserazid and carbidopa to ensure its bioavailability in the CNS and thereby its clinical effectiveness in patients (161). However, it does not relieve other disabling symptoms of PD possibly derived from alterations in other neurotransmitter systems, such as cholinergic, noradrenergic, and serotonergic systems (162, 163). Furthermore, several classifications of PD subtypes (164) were created with the need of an individual and/or combined strategy to pharmacotherapy during the course of the disease.

The optimal time for starting levodopa therapy in the clinical practice is still a matter of debate. Its short-term effect and long-term complications should be taken into consideration when planning the therapeutic strategies. Previous studies have shown that the choreiform dyskinesias occur in 17–45% patients and end-of-dose-wearing off in 37–57% patients in 2–5 years after the onset of levodopa therapy (165, 166). Even though the pharmacokinetic profile of levodopa is often blamed as the source of these complications, the exact cause is still far from understood (167). With conventional formulation of L-DOPA, irregular absorption and rapid catabolism are the basis of these long-term complications, which triggered the development of various other techniques including sustained-release oral formulations (161). Even though the correlation between L-DOPA therapy and the occurrence of long-term side effects was apparently stated as essential in many studies, the recent view considers the clinical phenotype and the individualized course as an even more important factor (168). A possible toxic nature of dopamine by accelerating the loss of dopamine neurons emerged earlier and could not be excluded appropriately (169). Thus, the lowest dosage providing satisfactory effect should be applied for the optimal output. In advanced stages of PD, there are evidence-based recommendations of strategies for providing more continuous dopaminergic stimulation or to offer DBS to the patients (170). However, drug adjustment is often considered based on the phenotype and clinical complications of the individual patient before addressing the indications for DBS. Recently, additional therapeutic options like fluid L-DOPA formulation or amantadine, safinamide and opicapone have been introduced (171–173).

## Clinical aspects of DBS surgery

### Preoperative management

DBS is an aggregate and interdisciplinary decision between patients, their families, neurologists, neurosurgeons, psychiatrists and neuropsychologists. All those involved in the process including patients themselves need to have realistic expectations after surgery. Patients should be made fully aware that DBS is not able to cure the disease but it is able to optimize mainly the motor symptoms, henceforth, the quality of life.

A detailed initial evaluation is needed in a movement disorder center to determine whether the patient will benefit from DBS. For this purpose, an experienced team of neurologists specialized in movement disorders and in particular in DBS, functional neurosurgeons, psychiatrists and psychologists with experience in movement disorders needs to be homogenized. As the first step, the diagnosis of idiopathic PD should be confirmed as other Parkinson syndromes usually do not respond to DBS (174). Furthermore, patient's current and past antiparkinsonian medication as well as the dosing schedule should be carefully reviewed. Subsequently, the response to dopaminergic medication (levodopa) should be (re)tested as the improvement of motor symptoms after the L-DOPA challenge is one of the very few known predictors of the clinical outcome after DBS (175). There is still an imperative need for the development of an objective and investigator-independent paradigm that can accurately denote the symptoms that could be targeted by DBS and the approximate improvement after the surgery (176). Several further clinical parameters have been analyzed as possible predictors of the post-operative clinical outcome of DBS-STN but until now dopaminergic response has been the strongest prognostic factor of post-operative outcome (177). Recently, the newly developed network and cortical morphometric parameters have also shown some promising results for the prediction of clinical outcome after the STN-DBS (86, 176).

In everyday clinical practice, the Unified Parkinson's Disease Rating Scale (UPDRS) score of a patient is assessed in the morning after overnight (approximately 12 h) withdrawal of levodopa and 20–60 min after the patient has ingested 1.5 times their normal morning levodopa dose. The best possible ON time is rated as ascertained by the patient and the examiner. Different dopamine agonists have different criteria to be paused before testing as mentioned in Table 1. Although there is no fixed limit of improvement required after the dopaminergic challenge for a DBS candidate (177), motor improvement of at least 30% is an objective response criterion (175). Conventionally, DBS is only offered to the patients who fulfill this response criterion because only those symptoms which are improved by levodopa are expected to be improved by DBS.

**Table 1:** Medication reduction scheme before L-DOPA challenge.

**Withdrawal scheme**	**Medication**
Overnight withdrawal (12 h)	Levodopa/Benserazide Levodopa/Carbidopa Levodopa/Carbidopa/Entacapone Entacapone Amantadine Safinamide Selegeline, Rasagiline
Withdrawal >24 h	Opicapone
Withdrawal >48 h	Pramipexole, Ropinirole, Rotigotine
Withdrawal >72 h	Cabergoline

There is no clear consent on the influence of age and disease duration on the post-operative outcome. In some studies, it has been shown that age and disease duration are not predictive for the post-operative motor outcome (177), while in others, it was shown that younger patients had more benefit from the DBS (178). Even though the reason why younger patients have better motor outcomes after DBS is not entirely clear, it might be that the older patients may have more comorbidities and less capacity for neuroplasticity. Similar outcomes have been shown in recent studies that included patients with early motor complications or with therapy refractory symptoms (179). A further important clinical criterion for a positive DBS response is the cognitive status of the patient. The presence of significant cognitive impairment is considered a contraindication for STN-DBS (180). A recent study using MRI demonstrated that cortical thickness of the frontal lobe (paracentral area and superior frontal gyrus) predicted the clinical improvement after STN-DBS. Moreover, in patients with cortical atrophy of these areas, a higher stimulation voltage was needed for an optimal clinical response (176). It has also been shown that bilateral STN-DBS is likely to have a negative impact on various aspects of frontal executive functioning (181). An exact preoperative assessment of the cognitive status by a neuropsychologist with experience in movement disorders should identify psychiatric symptoms such as psychosis, apathy, depression and anxiety and treat them optimally before considering or denying the DBS treatment. The possible cognitive and psychiatric side effects of medications (cholinergic agents, dopamine agonists, overdosed dopaminergic drugs) should be ruled out. For the cognitive assessment one of the following tests are conventionally used—Mini Mental State Examination (MMSE), Montreal Cognitive Assessment (MoCA), DemTect (182), Parkinson Neuropsychometric Dementia Assessment (PANDA) or Mini Mental Parkinson (MMP).

### Perioperative management

From the surgical point of view, eligible candidates for the DBS procedure should be in a satisfactory general and cognitive condition. Even though there is no difference in motor function outcomes for performing the surgery awake or under general anesthesia (183), most of the surgeries are realized with patients awake in order to perform intraoperative neurological tasks. In PD patients, concomitant disorders concerning blood clotting abnormalities, cardiovascular diseases and immune deficiencies should be evaluated. As a variety of disorders need anticoagulation therapy, patients should discuss with their general practitioners to determine the best perioperative bridging. Cardiovascular risks are discussed with the neuro-anesthesiologists and the immunological state of the patient should also be carefully considered, as post-surgical infection may lead to the ex-plantation of the DBS system.

Concerning the implantation of the DBS system itself, accuracy and safety are the most important factors to ensure a good patient outcome. Meticulous trajectory planning to avoid sulci, vessels, and ventricular walls is mandatory to minimize the risk of intracerebral hemorrhage. For the precise implantation, optimized imaging protocols with preoperative 3T magnetization-prepared rapid gradient-echo (MP-RAGE) MRI with low degree of distortion, use of contrast media for vessel visualization and modified T2-weighted sequences are used (184). Frame positioning, computed tomography (CT) scanning with the localizer and image fusion of the CT with the preoperative MRI may also influence electrode targeting. One of the issues unfavorably affecting the accuracy is the low rigidity of the implanted permanent electrode itself. Hence, intraoperative microrecording and macrostimulation is performed in many centers (most commonly in Europe) to confirm the optimal location of the electrode, and to overcome the brain shift problem due to liquor loss after opening the dura mater (185, 186). Implantation of the impulse generator on the same day or a few days after electrode implantation is the preference of the implanting center. After the surgery, a post-operative CT or MRI under safety recommendations of the manufactures is performed; monitoring in an intensive care unit is only required in case of intraoperative irregularities or post-operative delirium. Mobilization of the patients starts the day after the surgery. Regular wound inspection is necessary and patients are taught to leave all dressings in place and not to manipulate the wounds. The stiches are removed 10–12 days after the surgery. Intraoperative and post-operative scanning/imaging is performed to exclude electrode malpositioning and surgical complications (especially intracranial hemorrhage). Dopamine is given as soon as possible after the operation (if necessary over a gastric tube), with an approximately 25–50% reduction in the preoperative dosage due to a microlesioning effect (might be unstable) in the initial weeks.

Some patients develop post-operative confusion, which in most cases needs a supportive therapy with parenteral fluid therapy and reduced antiparkinsonian medication. In case of prolonged confusion, atypical antipsychotic agents, e.g., quetiapine, clozapine are used.

### Post-operative long-term care management

There is no consensus for starting the DBS programing but beginning the programming sessions a few weeks after the implantation allows time for reducing the microlesioning effect (187). As of late, stimulation based on constant current are applied in severe cases, as it makes the stimulation intensity independent of the impedance (188).

To start the stimulation, the neurologist performs a primary testing, checking the clinical effects and the therapeutic window (threshold determination for the clinical effects and the threshold for the side-effects) for each of the contacts and the range causing no side effects at each electrode contact, keeping the pulse width and frequency constant. The contact with the best clinical benefit/side effects ratio is then activated on both sides. The medication therapy is then adapted to the stimulation, the most common being the first levodopa monotherapy (189). The reduction of the L-DOPA needs a cautious approach to reach the threshold for best motor outcome with no troublesome dyskinesia. If the levodopa dose is insufficiently reduced, patients might develop side effects like dyskinesia or choreiform hyperkinetic movements, impulsivity and mania but on the other hand, reducing levodopa too much and too quickly might lead to apathy, depression or anhedonia (85). Moreover, it has been shown that decreased levodopa dose is a risk factor for developing restless leg syndrome (190). The efficacy of both DBS and dopaminergic therapy depends upon various factors such as the DBS target and dose of the medications. However, various cognitive symptoms such as the phonological and semantic verbal fluency, visuomotor processing or mood disorders (apathy, depression, anxiety) and other non-motor symptoms do not respond well to either therapies (DBS and L-DOPA) in a majority of patients (191). By adjusting the combined therapy, the neurologist should pay attention to the above-mentioned synergistic and diverse effects of the two therapies and the individual needs of the patients. Further decisions on medication therapy should be made according to evidence-based recommendations (170).

In addition, patients need specialized neuro-rehabilitation after DBS implantation (5). Before selecting a proper setting of post-surgical rehabilitation, the individual needs and goals for rehabilitation have to be defined for each DBS patient individually. The goal-specific therapy could be physiotherapy, Lee Silverman Voice Treatment (LSVT-BIG) therapy, speech therapy, occupational therapy, talk therapy, cognitive therapy or behavioral therapy depending upon the patient's need for best long-term clinical outcome (5).

## Discussion and conclusion

L-DOPA and DBS are now standard evidenced-based treatments for PD. Both improve motor symptoms in similar magnitudes, but show differential effects on dyskinesia, non-motor outcomes and activities of daily living. After an initial so called “honeymoon” period of L-DOPA, several limitations become apparent including postural abnormalities, freezing episodes, speech impairment, autonomic dysfunction, mood and cognitive impairment. Additionally, drug-related side effects especially psychosis, motor fluctuations, and dyskinesia are also observed in the long term (4, 192, 193). Similarly, DBS also fails to drastically improve the non-motor symptoms that significantly impact the quality of life of the patients (194, 195). A recent meta-analysis demonstrated that while there was similar individual efficacy of STN-DBS and L-DOPA, their combined effect on motor severity was additive within and beyond 5 years of follow-up (196). In support of the currently prevalent paradigm of reducing the dopaminergic tone during post-surgical management, it has been also shown that a lower reduction in dopaminergic medications might also result in a lower incidence of apathy and depression (197). Hence, the combination of both therapies irrespective of their differential mechanisms and outcomes might be the best approach until there is a clear understanding of the pathophysiology. However, a few recent studies have shown promising preliminary results in offering carefully selected PD patients earlier DBS treatment and delaying the severely disabling L-DOPA adverse effects (179, 198). Therefore, the time point of application of DBS is still a matter of debate and hence the subject of future studies to prolong the long-term benefits and to modify the natural disease course.

With the development of advanced imaging techniques and the availability of up to 7T MRI scanners, it will become possible for more accurate DBS. Moreover, new methods such as DTI will enable visualization of the white matter tracts that are close to the active DBS contact, and likely will provide a deeper insight into DBS mechanisms (199, 200). Furthermore, with the closed loop and adaptive stimulation techniques being developed, the dynamic conditions of stimulations might significantly reduce side effects of DBS (201, 202). Similar advancements in dopaminergic therapy include the improvement of a pump device for infusing L-DOPA in the jejunal cavity (203), approval of long-acting (5- to 6-h duration of action) L-DOPA (204), the new reversible MAO B-inhibitor safinamide to enhance the action of L-DOPA (205) and an extended-release (24-h long-acting) formulation of amantadine to markedly reduce the severity and extent of L-DOPA-induced dyskinesia (206). These developments will not only enhance the quality of life of the patients but also will aid in understanding the mode of action.

Dopaminergic drugs act on receptors not only in the nigrostriatal but also in the mesocortical and mesolimbic systems. This characteristic distribution of different receptors ensures its general effect on brain networks and explains its side effects. The advantage of DBS over dopaminergic therapy is that it acts on selective anatomical networks. The neuroanatomical selectivity of DBS warrants less possibility of stimulation-evoked side effects, especially with the recently implemented directional stimulation through segmented electrodes. However, even though the neuroanatomical selectivity of DBS is a huge benefit, medication therapy and–even more so–the combination of both is still of utmost importance for the best clinical outcome for a multisystem disorder like PD. Detailed exploration of STN and GPi connections and their somatotopy, which is still missing in humans, would further enhance the outcome of dopaminergic and neurostimulation treatments. A meticulous pre-, peri-, and post-operative management is crucial for the best clinical outcome.

## Author contributions

MM and NK did the literature review and wrote the manuscript. DC and StG contributed for the review of clinical aspects of L-DOPA. BP and MG reviewed the clinical aspects of DBS surgery. GT and SeG contributed with the critical review of the article. All authors discussed the manuscript and agreed to the final version.

### Conflict of interest statement

The authors declare that the research was conducted in the absence of any commercial or financial relationships that could be construed as a potential conflict of interest.
